# Sustainable Biomass-Derived Carbon Electrodes for Potassium and Aluminum Batteries: Conceptualizing the Key Parameters for Improved Performance

**DOI:** 10.3390/nano13040765

**Published:** 2023-02-17

**Authors:** Glaydson Simões Dos Reis, Shaikshavali Petnikota, Chandrasekar M. Subramaniyam, Helinando Pequeno de Oliveira, Sylvia Larsson, Mikael Thyrel, Ulla Lassi, Flaviano García Alvarado

**Affiliations:** 1 Biomass Technology Centre, Department of Forest Biomaterials and Technology, Swedish University of Agricultural Sciences, SE-901 83 Umeå, Sweden; 2 Department of Chemistry and Biochemistry, Facultad de Farmacia, Universidad San Pablo-CEU, CEU Universities, Urbanización Montepríncipe, 28668 Madrid, Spain; 3 Institute of Materials Science, Universidade Federal do Vale do São Francisco, Avenue Antônio Carlos Magalhães, 510-Santo Antônio CEP, Juazeiro 48902-300, BA, Brazil; 4 Research Unit of Sustainable Chemistry, University of Oulu, P.O. Box 3000, FI-90014 Oulu, Finland; 5 Unit of Applied Chemistry, University of Jyvaskyla, Kokkola University Consortium Chydenius, Talonpojankatu 2B, FI-67100 Kokkola, Finland

**Keywords:** biomass-carbon anodes, biomass-carbon cathodes, potassium battery, aluminum battery

## Abstract

The development of sustainable, safe, low-cost, high energy and density power-density energy storage devices is most needed to electrify our modern needs to reach a carbon-neutral society by ~2050. Batteries are the backbones of future sustainable energy sources for both stationary off-grid and mobile plug-in electric vehicle applications. Biomass-derived carbon materials are extensively researched as efficient and sustainable electrode/anode candidates for lithium/sodium-ion chemistries due to their well-developed tailored textures (closed pores and defects) and large microcrystalline interlayer spacing and therefore opens-up their potential applications in sustainable potassium and aluminum batteries. The main purpose of this perspective is to brief the use of biomass residues for the preparation of carbon electrodes for potassium and aluminum batteries annexed to the biomass-derived carbon physicochemical structures and their aligned electrochemical properties. In addition, we presented an outlook as well as some challenges faced in this promising area of research. We believe that this review enlightens the readers with useful insights and a reasonable understanding of issues and challenges faced in the preparation, physicochemical properties and application of biomass-derived carbon materials as anodes and cathode candidates for potassium and aluminum batteries, respectively. In addition, this review can further help material scientists to seek out novel electrode materials from different types of biomasses, which opens up new avenues in the fabrication/development of next-generation sustainable and high-energy density batteries.

## 1. Introduction

The widespread development of portable and wearables electronics, plug-in electric vehicles, and the requirement of large-scale production of smart grids [1] are the driving force for developing more efficient energy storage devices beyond lithium-ion batteries (LIBs) which [2] represents a bottleneck in the state-of-the-area for batteries due to its earthly scarcity (only 0.0017 wt.% of earth crust) and thence casts as ¨white gold¨. In pursuit of alternatives to LIBs, the development of electrochemical devices based on sodium, potassium, and aluminum are considered promising steps for the increasing production of batteries with low cost. With an estimated total concentration of sodium (2.8 wt.%), potassium (2.6 wt.%), and 8.1 wt.% of aluminum in the earth’s crust [2], they are considered an important component for shuttle-carrier in rocking-chair type barriers for stationary (off-grid) storage applications where the battery size doesn’t matter. In the case of sodium, recent developments have reached their path to the market even though Na^+^ diffusion has been sluggish in many hosts [3,4,5]. 

The principle of operation for potassium-ion batteries (KIBs) is based on the commutation of K-ions between the cathode and anode during cycling, in a process characterized by slow diffusion kinetics in response to the large K-ions [6]. An important key parameter for KIBs refers to the intercalation of the K-ions in graphite [1] that results in the formation of KC_8_ with stage-1 structure, improving its electrochemical performance when used as a negative electrode for 4 V class KIBs [7,8].

On the other hand, aluminum batteries (ABs) function based on two separate interfacial reactions in-between anode-electrolyte and cathode-electrolyte. A typical AB comprises of graphite cathode and AlCl_3_-ionic liquid (IL) electrolyte that could deliver experimental capacities as high as 100–150 mA h g^−1^ [9,10]. The electrolyte reaction between AlCl_3_ and ILs such as 1-ethyl-3-methylimidazolium chloride ([EMIM]Cl) creates complex chloroaluminate species, AlCl_4_^−^ and Al_2_Cl_7_^−^, among which the former one mainly serves as charge carriers in the cathode. During the discharge, AlCl_4_^−^ deintercalated from the graphite cathode and interacts with the Al anode to form its dimer Al_2_Cl_7_^−^ [9,10]. These reactions readily take a reverse course upon charging and that is how secondary or rechargeable ABs work. Graphite is the most widely studied current AB cathode which undergoes huge volume expansion (~600%) upon AlCl_4_^−^ (6.09 Å) insertion into its limited *d*-spacing (3.35 Å) [9,10]. The large size of the anionic charge carrier accompanied by these adverse volume changes often induces structural damages leading to electrode failure and hence results in low experimental capacities. These are the two major limiting factors that mainly affect the performance of current ABs. 

To address these issues, many materials have been developed aiming to replace graphite in battery systems such as organic materials [11,12,13,14,15,16,17,18,19,20]. Among them, biomass-derived carbon materials are of great interest because of their sustainability, environmentally benign, and being reasonably inexpensive [21]. In addition, biomass/carbon materials possess well-developed porosity with high specific surface area, rich in micro- and meso- pores and abundant functional groups, excellent prerequisites for high-performance electrode materials [6,7,8,21]. However, the low electrochemical storage capacity is still a challenge for the practical application of biomass carbon materials for battery applications. Nevertheless, there are many strategies to improve biomass carbon properties for boosting its electrochemical performances [22,23,24].

Compared to other battery systems such as lithium and sodium, there are very few systematic summaries reporting on the biomass carbon materials’ utilization in KIBs and none in ABs. Therefore, it is urgent to summarize some important development in biomass carbon materials in KIBs and ABs. There are still important challenges for the productivity of biomass-based carbon materials due to the complexity and uneven chemical components (lignin, cellulose, hemicellulose) of the different biomass precursors that vary from each one of them depending on the sources [25]. Considering that different biomass precursors, as well as preparation conditions, can generate carbon materials with extremely different physicochemical and electronic properties, many parameters should be taken into account for the preparation of the carbon materials electrodes such as an appropriate pyrolysis set-up and chemical activation as well as a complete knowledge of the biomass precursors in terms of its main constituents [22,23,24,25].

The development of biomass-based materials acting as carbon derivatives with a highly porous surface introduce benefits due to the simple, low cost, and massive production in association with the simple incorporation of heteroatom doping elements. This process contributes to the diffusive transport of large ions and the incorporation of catalysts into metal-free electrodes for batteries. 

This perspective reviews the different strategies for the employment of biomass residues as sustainable and efficient precursors to fabricate carbon anodes for potassium and aluminum batteries. It mainly focuses on the relationship between the composition of biomass precursor and synthesis method to physicochemical properties of biomass-derived carbon nanostructure over their electrochemical performances in potassium and aluminum batteries. Some important challenges and future works are also addressed in this report.

## 2. Biomass-Based Anodes for Battery Application

Graphite, the carbon allotrope, with a layered structure is the most successful commercialized anode for LIBs. No more than six carbon atoms cling to one Li-ion forming LiC_6_, which amounts to a theoretical capacity of 372 mA h g^−1^ [26] pulling it down for application in high-energy devices such as plug-in electric vehicles. Moreover, employing graphite as an anode for other battery configurations: (a) for Sodium ion battery (SIB) is not feasible as the graphitic intercalating compound, NaC_6_ is thermodynamically unstable unlike other battery chemistries and hence, hard carbon is used as a potential SIB anode; (b) for potassium battery, though the intercalating KC_8_ phase is stable but limited in their specific capacity due to their large K atomic radii (280 pm) accompanied with huge volume change during cycling. To overcome these limitations, significant efforts have been focused on biomass-carbon anodes (amorphous and non-graphitic bio-based carbons) for battery application, which often outperform the electrochemical performances of graphite as anode [27,28].

In addition, biomass-derived carbon anodes have advantages such as biomass precursor’s widespread availability, outstanding electronic conductivity, low cost, and favorable hierarchical physicochemical properties for insertion/de-insertion of K^+^ and AlCl_4_¯ in respective KIBs and ABs. An important dependency of using biomass-derived carbonaceous anodes is that these materials are synthesized from any type of biomass residues and could be tailored to have certain electrochemical properties adjustable to boost their anode performances that are suitable for potassium and aluminum-based batteries [29]. The important requirements for biomass carbon materials to be suitably reversible for potassium and aluminum batteries include: (i) the ability to insert/extract K/K^+^ and AlCl_4_¯ in the carbon matrix at the low potential to have a stable build-in solid-electrolyte-interface (SEI) protective layer; (ii) intercalate/de-intercalates reversibly more K/K^+^ and AlCl_4_¯with negligible volume change to boost specific capacity and energy density with long cycle life. 

The physicochemical versatility structuring of biomass-derived carbon anode materials makes them extremely suitable for energy storage systems. The biomass-derived carbon anode materials properties can be tailored to have desired properties for boosting its electrochemical properties such as (i) high specific surface area (SSA) and developed hierarchical pore structures containing macro/meso/micropores that can maximize the charge/ion storages and (ii) large interlayer spacings that boost the K^+^ and AlCl_4_^−^ insertion into the anode microstructure and also provide good mechanical stability for the anodes during the ion insertion/extraction process; (iii) materials with highly active surfaces due to the presence of a high number of functional groups on their surfaces, which enable chemical reactions that can boost K^+^ and AlCl_4_¯ transfer; and (iv) easily modified by introducing more electrochemically active elements such as heteroatoms (N, O, F, H, S, B, etc) that can further improve its physicochemical characteristics and henceforth their electrochemical performance.

## 3. Effect of Preparation Conditions on Biochar Physicochemical Properties

**Figure 1:**
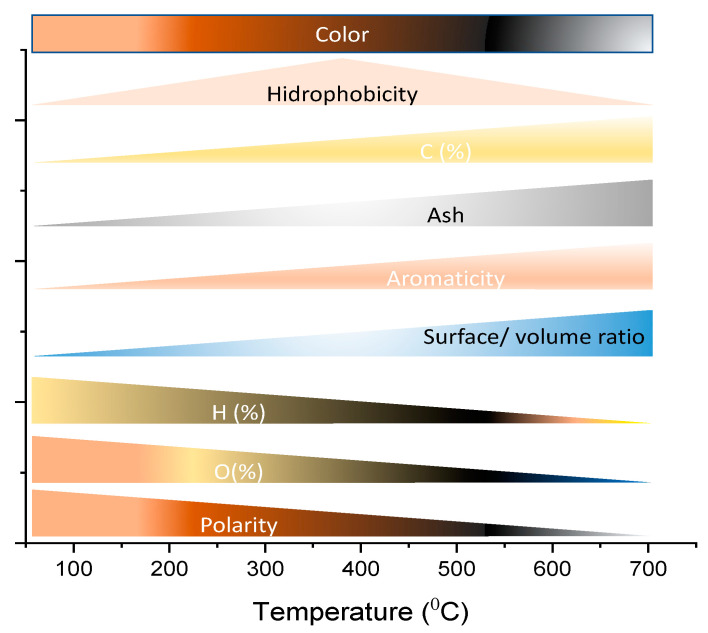
Variation in biochar properties with an increase in pyrolysis temperature conditions Adapted from Ref. [35].

The literature shows that the properties of biochars are severely dependent on the pyrolysis conditions in which they are prepared [22,27,30,31,32,33,34]. Figure 1 summarises the main characteristics of the biochars concerning the pyrolysis temperature; these characteristics are of significant importance for biochars that are to be considered anodes for battery applications. Of course, other pyrolysis parameters (biomass composition, reaction time, catalyst, heating rate, and atmosphere) must also be taken into account since they often influence concomitantly. 

Figure 1 shows that even the color of the biochar can be influenced by the pyrolysis temperature (that turns from brown to black as the temperature increases). The carbon (as well as aromaticity) and ash content also can increase with the increase in temperature depending on the type of biomass precursors and pyrolysis atmosphere. It is very important to synthesize biochar with a high percentage of carbon and minimal/or zero ash content. Elevated carbon content is quite often related to good biochar with physicochemical, electrical, and thermal characteristics while the ash is often responsible for decreasing SSA values and functional groups on biochar’s surfaces, which are crucial properties for an efficient biobased electrode for battery applications. For instance, dos Reis et al. [30] used ZnCl_2_ as an activating agent to prepare biochars with high carbon content >93% from spruce bark biomass as compared to biochars with a reduced carbon content of 87.8% and 82.7% as reported, respectively, by Correa et al. [31] and Duan et al. [32].

The specific surface area (SSA, in m^2^ g^−1^) of biochars quite often obeys the trend of increasing their values with the increase in pyrolysis temperature; however, it also depends on the biomass source, the catalyst used, and other pyrolysis parameters. For instance, dos Reis and co-workers reported [33,34] using different activation agents (KOH and ZnCl_2_) to synthesize high SSA biochars before subjection at high pyrolysis temperatures, respectively, 700 °C and 900 °C. The KOH-activated biochar exhibited a high SSA of 1881 m^2^ g^−1^ as compared to ZnCl_2_-activated biochar (1294 m^2^ g^−1^). However, if the temperature increased >1000 °C, the SSA values tend to decrease because of shrinkage of the biochars narrowing their pores leading to collapsing their meso/microporosity at such extremely high temperatures.

In another work [36], dos Reis et al. exhibited the synthesis of biochars with different physicochemical properties depending upon the chemical activating agents such as KOH, ZnCl_2_, ZnSO_4_, and MgCl_2_. They conclude by stating that KOH yielded a biocarbon with a high SSA (2209 m^2^ g^−1^), followed by biochars using ZnCl_2_ (1019 m^2^ g^−1^), ZnSO_4_ (446 m^2^ g^−1^), and MgCl_2_ (98 m^2^ g^−1^) while latter activation produced a hydrophobic material than other activators. The carbon and oxygen content were 86.6% and 10.5%, 94.7% and 4.4%, 95.6% and 3.0%, and 97.0 and 2.7%, for KOH, ZnCl_2_, ZnSO_4_, and MgCl_2_, respectively.

Gonzalez-Hourcade et al. [37] reported the production of nitrogen-doped bio-based materials with improved properties. Microalgae were used as carbon precursors, melamine was employed as a nitrogen source, and H_3_PO_4_ as a chemical activator. It was concluded that the nitrogen doping process increased the SSA by 33% as well as the number of micropores in the carbon structure. In addition, nitrogen doping helped to increase the graphitization degree, the number of oxygen species and nitrogen groups on the biochar’s surface, which can further boost its electrochemical performances.

Based on a carbon-silicon systematic study, Tang et al. [38] reported that the oxygen content plays a key role in the performance balance of anode materials. Peng et al. [39] studied the effect of oxygen functionalities on the regulation of carbon/electrolyte interfacial characteristics toward K^+^ charge storage. The authors found that such functionalities boosted the K^+^ storage performance of carbonaceous materials. It was revealed that the C=O and COOH rather than C-O-C and OH groups strongly contributed to K^+^ storage capacity. Using in-situ FT-IR spectra and in-situ electrochemical-impedance-spectroscopy (EIS), it was reported that the oxygen-containing functional groups regulate the components leading to the formation of highly conductive, intact and robust SEI. Jerigová et al. [40] stressed that surface oxygen functionalities are responsible for enhancing the capacity of carbon electrodes in metal-ion batteries; mainly because these functionalities provide a uniform metal (Li^+^, Na^+^, K^+^ and AlCl_4_^−^) deposition on carbon surface without dendrites formation. 

Heteroatom doping is widely employed to be an effective strategy to significantly enhance the electrochemical performance of carbon electrodes; this is mainly because the insertion of heteroatoms can create surface functionalities, create defects, enhance the conductivity, improve the porosity, or enlarge the carbon interlayer spacing [41,42,43]. The insertion of heteroatoms in the carbon structure provokes electron density changes, which may change the polarity of the carbon surface, generating binding sites formation ions/anions, and making the surface catalytically active for different reactions including in batteries [44]. Heteroatoms such nitrogen, boron, phosphorous, and others can act as an electron donor that gives more electrons to the delocalized carbon network, increasing the electronic conductivity and therefore, boost the Li^+^ and Na^+^ storage capabilities. In addition, heteroatom doping may expand the carbon lattice, which provide more space to charge accommodation, therefore boosting the ions storage and the battery performance [45]. 

Another anode property that is reported to influence the electrode electrochemical performance is the hybridization content (sp^2^/sp^3^ ratio), because this ratio determines the atomic arrangement of the carbon structure [46]. Carbon materials rich in sp^2^ content is related to graphitic stacking, while sp^3^ is related to carbon structures with no layers, disordered and amorphous [47]. The sp^2^–sp^3^ hybridization state are important parameters governing the final anode materials’ stability, reactivity, and physical properties [48]. Lu et al., [46] employed an anode based on dodecahedral carbon framework, and studied the level of its electronic structure (sp^2^/sp^3^ hybridized orbital) on their corresponding Li-ion storage performances. The experimental capacity of the anode exceed its theoretical capacity by 298 mA h g^−1^ during 250 cycles at 0.1 A g^−1^. The authors stated that the extra capacity was due to the non-coplanar sp^2^ /sp^3^ hybridized orbital controlling non-Euclidean geometrical structure, which acts as new active sites toward the excess ion adsorption and charge storage.

The crystalline structure (graphitic structure) of the bio-based carbon anodes may also have influence on its electrochemical performances and on the K^+^ storage mechanism and performance [49]. A large presence of crystalline (graphitic) sheets give location for the occurrence of staging behaviour. Moreover, a stable graphitic structure can sustain a low working voltage plateau and give a high capacity at a low current density. Overall, layered crystals in biobased carbons are suitable for intercalation processes because they can strongly adsorb compounds into the van der Waals voids between each of their layers. Wang et al. [50] reported a correlation between crystallinity of carbon layers with SSA values, which then has huge influence on initial Coulombic efficiency (ICE) of batteries. It was reported that anodes with large SSA lead to a decrease in ICE, which is narrowly related to the electrolyte reduction at the anode’s surface, and that an increase in crystallinity composition of the anodes helps to reduce the contact area between the carbon layers and the electrolyte, thereby improving ICE.

## 4. Potassium-Ion Battery (KIBs)

The overwhelming demand for ‘white gold’ i.e., lithium, for the deployment of electric vehicles leads to high cost due to its earthly scarcity and whereabouts in politically unstable countries. Therefore, there is an urge to develop sustainable battery chemistries like potassium-ion batteries (KIBs) whose working principle is similar to LIBs though the different atomic properties introduce some limitations. The bigger size of K^+^ than Li^+^ makes the amount of K^+^ intercalated, in similar hosts, smaller. K^+^ has weaker Lewis’s acidity and smaller solvation ions size compared to Li^+^, thereby displaying an improved ionic conductivity and mobility which can exhibit faster kinetics and better charge transfer [51]. Considering it weighs higher, the expected practical specific capacity is lower; the standard reduction potential of K^+^ is −0.11 V lower than Li^+^ thereby making the specific energy lower. Therefore, KIBs are not presently competitive for EV applications but can replace LIBs in applications where the battery weight, volume/size is not a constraint, while the price is cheaper than lithium, since potassium can be considered a limitless resource. In addition, aluminum could replace expensive copper as an effective current collector would make it cost low and lightweight battery configurations, instead of copper (which is used as a negative electrode current collector for LIBs), aluminum which is cheaper and lighter than copper, can be used in KIBs, which makes this battery cheaper and also can provide higher gravimetric energy density of KIBs than that of LIBs. 

The large K^+^ radius (2.80 Å) may result in a significant volume variation of electrode materials during the K^+^ insertion and de-insertion process, therefore, the anode must have high SSA, pore size distribution, and suitable lattice sites/spaces to accommodate volume change and release the K^+^ freely in a reversible way to obtain high specific capacities. This is a very important requirement of biomass carbon to be efficiently employed as KIBs’ anodes to have efficient and long stable cycling performances. The other factors that affect the KIBs’ performance include the physicochemical characteristic of the biobased anodes. For instance, the rate performance is severely dependent on the anode reaction dynamics that in turn is influenced by the K^+^ diffusion and the electrical conductivity of the biobased anodes; these features are highly affected by the anode’s physicochemical characteristics. Yang et al. [52] synthesized a biomass KIB’s anode and reported excellent electrochemical metrics; a high specific capacity of 407 mA h g^−1^ at 50 mA g^−1^ and 163.8 mA h g^−1^ at 200 mA g^−1^ after 50 and 100 cycles, respectively. They stated that such metrics were achieved due to the presence of crystal structures of non-graphitic carbons with an enlarged interlayer distance that maximized the K^+^ accommodation. In addition, the biocarbon anodes with a versatile hierarchical structure of pores/defectives edges exhibited a high amorphous degree, which helped alleviate volume change; enabled the rapid ions-diffusion and improved electrolyte wettability of the anodes for easier K^+^ insertion/extraction and thereby improved their cycling performance.

**Figure 2:**
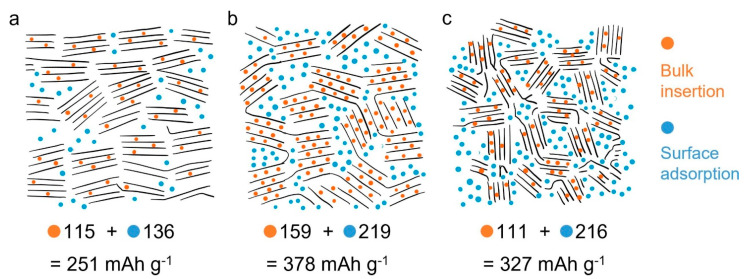
Proposed K-ion storage mechanisms in L700 (**a**), M700 (**b**), and H700 (**c**). Reprinted with permission from [53] Copyright 2022, Elsevier.

An important work published by Wu et al. [53], studied the effect of lignin molecular weight on lignin-carbon anodes on K^+^ storage mechanisms. The authors stated that the K^+^ storage in lignin anodes was highly dependent on the bulk-insertion and surface-adsorption sites, which in turn are influenced by pyrolysis conditions as well as the characteristics of lignin precursors (molecular weight). The K^+^ storage took place in the lattice interlayer spaces of the biocarbon anode structure. Further understanding the mechanism, the adsorption of K^+^ on the biochar surface was maximized on the disordered carbon areas of the lignin-based anodes. The authors stated that under pyrolysis treatment, the lignin precursor with low molecular weight generated biochars with graphite-like structures due to the easier depolymerization process at high temperatures that increases the degree of graphitization. However, because low molecular weight lignin-based biochars presented less disordered carbon structures, K^+^ adsorption is reduced which negatively impacts total K-ion storage capacity (Figure 2a). On the other hand, the lignin precursor with high molecular weight yielded biochar anodes with more disordered structures because long lignin chains are more difficult to be depolymerized, fragmented, and aromatized. The biochar anode with more disordered carbon structures increased the adsorption of K^+^ (Figure 2c). However, when the anodes were prepared from lignin with medium molecular weight, they exhibited a well-balanced combination of disordered and graphite-like structures, which in turn, boosted the K^+^ storage anode performance by combining surface adsorption and bulk-insertion charge storage mechanisms (Figure 2b).

We point out that the above findings can also be valid for preparing biomass-anode materials with a wide range of lignin content in their structures and for many other battery applications such as LIBs, SIBs, and aluminum batteries, limited to KIBs. Studying how the lignin content affects the biomass-anode structure and electrochemical performances can also be extended to a similar study, how the different biomass precursors (and their structures) influence their respective carbon anode materials’ physicochemical and electrochemical features. For instance, a representative selection of biomass precursors and their anodes’ morphological, structural, and electrochemical performance on KIBs are shown in Table 1. The table shows that the diverse biomass precursors generated different surface morphologies and porosity structures, which impacted their electrochemical performance. The physicochemical features of the biomass-derived anodes play a crucial role in its electrochemical metrics, due to the accumulation of ions at the micropores, diffusion, and storage of ions at the mesopores and macropores. The different hierarchical pores and surface properties enable the anodes to maximize their specific capacity and show positive routes for future developments.

**Figure 3:**
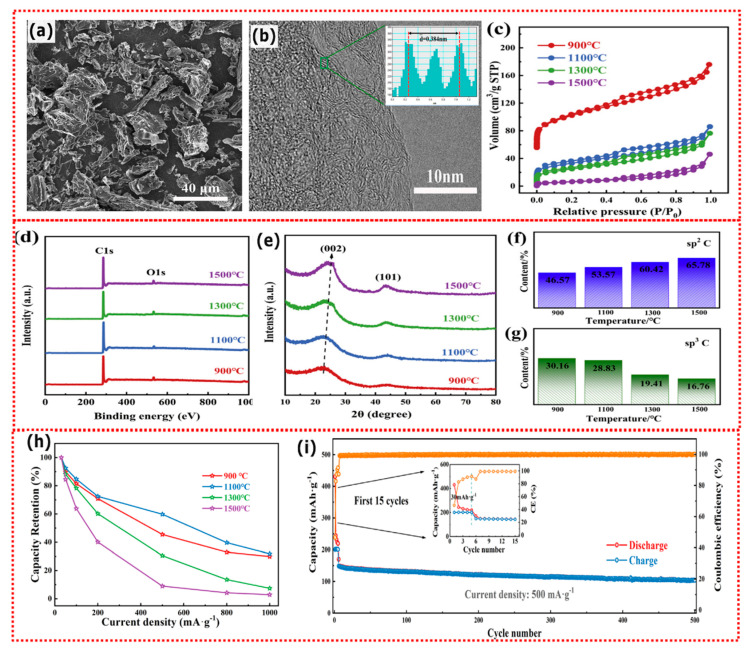
(**a**) SEM image of rice husk carbon anode; (**b**) TEM image of rice husk carbon anode; (**c**) N_2_ adsorption/desorption of rice husk carbon anodes; (**d**) XPS spectra of rice husk carbon anodes; (**e**) XRD patterns of rice husk carbon anodes; (**f**,**g**) Carbon states content as a function of the temperature of rice husk carbon anodes; (**h**) Comparison of capacity retention of KIB cells at increasing current densities among different carbon anodes; (**i**) Cycle performance of biochar with highest performance (1100 °C) at 500 mA·g^−1^ (inset: the first fifteen cycles). Reprinted with permission from ref. [54], Copyright 2020, Elsevier.

Li et al. [54] reported the preparation of porous carbon via a simple pyrolysis method of rice husk as biomass precursor. The biomass carbon materials were prepared at temperatures of 900, 1100, 1300 and 1500 °C (named RHC-900, RHC-1100, RHC-1300 and RHC-1500). Figure 3 summarises its characterization and application as KIB anodes. Figure 3a exhibited a broken morphology with no evident porosity while from the TEM image (Figure 3b), it can be observed that there are random and disordered graphite microcrystals, indicating a highly amorphous microstructure. The average interlayer spacing of biochar-anode was calculated to be 0.384 nm, which is larger than graphite (0.335 nm) and therefore favors the K^+^ insertion/extraction. XPS spectra (in Figure 3d) depict that the porous carbons are mainly composed of carbon (C 1s) and oxygen (O1s) states. The XRD patterns (Figure 3e) exhibit two broad characteristic peaks, corresponding to (002) and (101) planes for amorphous carbon with no evident crystalline phases.

The carbon materials were quantified in terms of carbon states sp^2^ (that represents the graphitized carbon) and sp^3^ (that is linked to the carbon defects) [55]. It was found that the sp^3^ peak area reduced while the sp^2^ increased as a function of temperature suggesting a higher degree of graphitization. These anode characteristics can be highly beneficial for the rate performance of the battery, including KIBs (Figure 3h). Between all anodes, the one pyrolyzed at 1100 °C showed the best rate capability, which retained 62.72 mA h g^−1^ at 1000 mA g^−1^. The better result of biochar-anode pyrolyzed at 1100 °C could be related to the bigger interlayer distance, which maximizes K^+^ insertion and its diffusion. Since RHC-1100 displayed better rate capability it was also subjected to the long cyclability tests and it delivered a capacity of 125.7 mA h g^−1^ at 200 mA g^−1^ after 300 cycles and 103.77 mA h g^−1^ at 500 mA g^−1^ after 500 cycles, suggesting the great practical application of biomass materials as KIBs anode.

Wang et al. [56] used corn husk as a carbon precursor to fabricate KIBs anodes via one-step carbonization. The biomass-derived carbon anodes exhibited a very small surface area (3.4 m^2^ g^−1^) but were rich in macropores, and even so have demonstrated electrochemical performance much better than many porous materials. At 100 mA g^−1^, the biochar anode delivered its first discharge and charge capacities of 396 and 231 mA h g^−1^, respectively, and keeping a reversible capacity retention rate of 89.1% after 100 cycles. Furthermore, after 500 cycles and at a higher current density (1000 mA g^−1^) it delivered a capacity of 135.3 mA h g^−1^. It was stated that such high electrochemical efficiency was mainly due to the presence of nitrogen and oxygen heteroatoms as well as the large interlayer spacing.

**Table 1:** Comparative electrochemical performance of biobased carbon material anodes for KIB.

Biomass Source	Synthesis Method zand Morphology	Specific Surface Area (SBET, m^2^ g^−1^)	Potential (V vs. K/K^+^)	Current Rate (mA g^−1^)	Initial Discharge Capacity (mA h g^−1^)	Capacity Retention (mA h g^−1^)/(cycles)	Ref.
Ganoderma lucidum spore	Anode prepared by one-step carbonization with no activation. Hollow-cage structure with no high porosity and a high degree of graphitization with a typical carbon amorphous structure.	104.4	0.02–3.0	1000	~450	~125 (700)	[52]
Rice husk	Carbonization temperatures from 900 to 1500 °C. The highly amorphous microstructure is rich in mesopores.	365	0.01–3.0	30	~250	104 (500)	[54]
Bamboo	Carbonization and KOH-treated activation. Medium porosity carbon anodes with crystalline structures with a high presence of defects and low graphitization degree.	339	0.01–2.8	50	~450	204 (300)	[57]
Sugarcane bagasse	Biomass activated with NiCl_2_. Highly amorphous and microporous materials doped with nitrogen.	~467	0.1–3.0	100	142	100.4 (400)	[58]
Chitin	NaOH/urea activated carbon anodes. Meso-macro porous chitin microspheres-like materials	563	0.01–3.0	0.12 to 36 Coulombic	~320	180 (4000)	[59]
Corn silk	Hydrothermal treatment and one-step carbonization. The carbon materials possess larger lattice spacing, amorphous structures, and a very low degree of graphitization with many defects.	-	0.01–3.0	100	~886	~121 (2600)	[60]
Hemp core	One-step carbonization with fluorine doping process. Material wrapped by fluorine-containing nanotubes rich in defects with wide pore sizes and a low degree of graphitization.	780	0.001–3.0	200	~822	369.6 (500)	[61]
Maple leaves	Carbonization and HNO_3_-treated activation. Low porosity carbon anodes containing O/N functional groups with higher graphitization degree.	62.6	0.01–3.0	50	~934	~142 (1000)	[62]
Cyanobacteria powder	Two-step carbonization with NaCl and KCl activation. Nitrogen/oxygen co-doped hierarchically mesoporous carbon. Amorphous structures with a high degree of graphitization.	473	0.01–3.0	50	912	~104 (1000)	[63]

**Figure 4:**
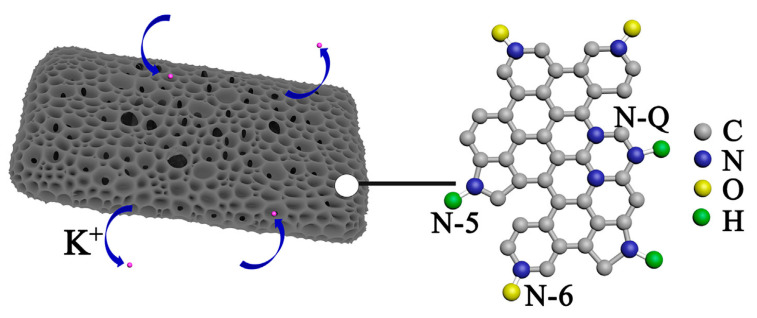
Proposed mechanism of K-ion storage in non-porous biochar. Reprinted with permission from [56]. Copyright 2017, Elsevier.

The high presence of heteroatoms such as O, and N provided more active sites of K^+^ storage (see Figure 4). The type of interlayer spacing facilitated the intercalating of K^+^ into the graphite layers. Moreover, the non-presence of micro-mesopores (low porosity) and the high presence of macropores resulted in a very high Coulombic efficiency (Figure 4). These findings highlight that the SSA is not always the main parameter for boosting the anode electrochemical properties but also the surface functionalities and element chemical states present in the anode nano/microstructures [56].

## 5. Aluminum Battery

**Figure 5:**
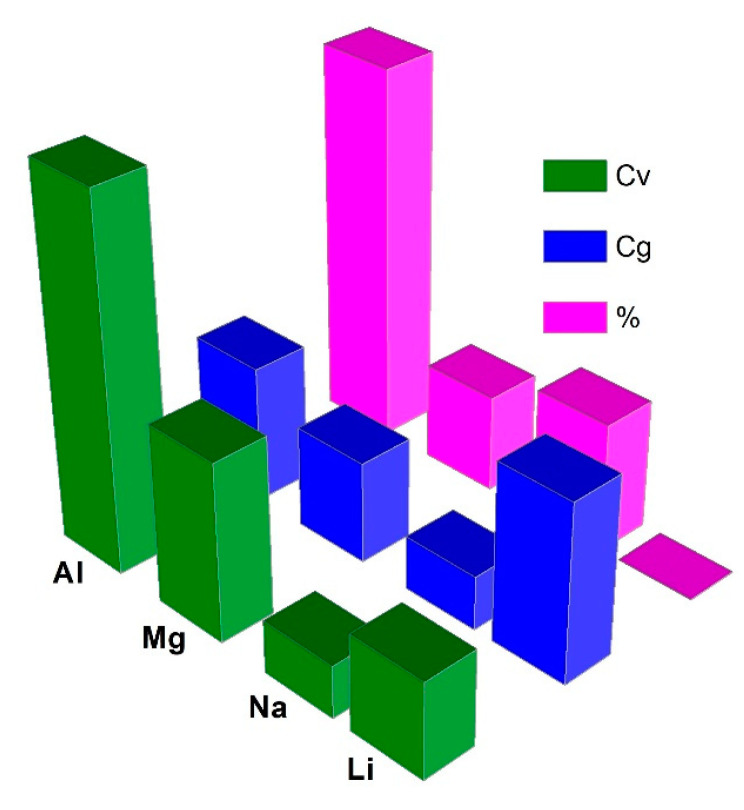
C_v_, C_g_ and % abundance comparisons of Li, Na, Mg, and Al.

Aluminum battery (AB) has also garnered much attention lately mainly due to their low price, rich natural ingredients, and safety. For instance, Al is the most abundant metal (82,000 ppm) as compared to Li content of 18 ppm on the earth’s crust. Another important aspect of AB is that Al featured three-electron redox properties (Al^3+^/Al), which may sustain very high theoretical gravimetric (C_g_ ~ 2980 mA h g^−1^) and volumetric (C_v_ ~ 8046 mA h cm^−3^) capacities [64,65,66,67]. These values are some of the highest known for battery materials as compared in Figure 5 and Table 2 [67]. Another important advantage of AB is that Al presents low reactivity towards the air and moisture compared to hyperactive Li and therefore it can be handled in ambient conditions of large-scale battery fabrication facilities such as dry-room. Most of the ABs utilize AlCl_3_-ionic liquid (IL) electrolytes that bear high autoignition temperatures (>300 °C) and ensure further safety as an in-built feature when compared with hyperactive and less ambient temperature tolerant LIBs (<60 °C) [68]. Furthermore, the IL electrolytes endure over several hundred thousand cycles without any chemical changes [10,65,69,70]. ABs could be a better choice for stationary applications owing to their low flammability and longer lifespan similar to lead-acid batteries [65,66,68] Furthermore, cheaper economic prospects of Al (US$~2.4/kg vs. US$~75.0/kg for LiOH·H_2_O; for pure Li price is ~50× more) [71,72,73,74] comparable to gasoline (US$~1.57/kg; US$~4.467/gallon) [75] are favorable for developing electric vehicles (EVs) those eventually drives away our society from heavily dependent on fossil fuel transportation needs. These important advantages highlight AB as low-cost energy storage devices with high capacity and improved safety [76].

**Figure 6:**
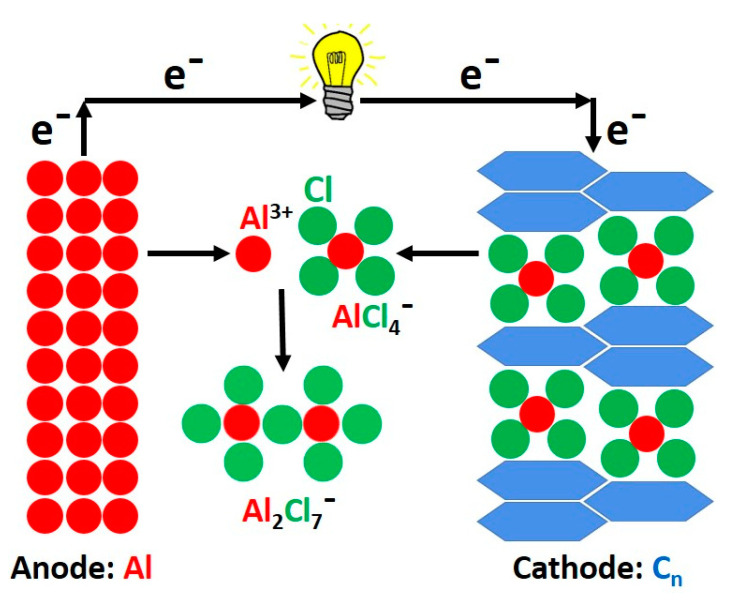
AB discharging in typical AlCl_3_—IL electrolytes.

The working principle in ABs is based on intercalation/deintercalation chemistry, where two interfacial reactions take place with AlCl_3_-IL electrolytes. The two half-cell reactions taking place on the anode and cathode sides are shown in Equations (1) and (2), respectively. Firstly, on the anode side, electrochemical deposition and dissolution processes occur between Al metal and electrolyte take place as described in Equation (1); secondly, at the cathode (graphite or graphene, C_n_) side, where intercalation/deintercalation processes of AlCl_4_^−^ anions take place during the charge/discharge process as described in Equation (2) [64,65,66]. The operating principle of a typical AB is shown in Figure 6. The complex two interfacial reaction chemistry of ABs categorizes them into dual-ion (anion) batteries (DIBs) and not Al-ion batteries (AIBs) similar to LIBs. However, due to the reversible electroplating process of Al metal at the anode, it is possible to employ IL electrolytes, generally composed of a eutectic mixture of AlCl_3_ and an organic chloride salt from the alkyl-imidazolium halogenides such as 1-ethyl-3-methylimidazolium chloride ([EMIM]Cl). However, finding a suitable electrolyte-cathode pair for AB remains one of the biggest challenges to overcome. But in this review, the issues involving electrolytes will not be addressed, rather the issues involving the cathode side.

(1) $$Anode:\;\mathrm{Al}+7{\;\mathrm{AlCl}}_{4}^{-}⇋\;4{\;\mathrm{Al}}_{2}{\mathrm{Cl}}_{4}^{-}+3{\;e}^{-}\;$$

(2) $$Cathode:{\;C}_{n}\left[{\mathrm{AlCl}}_{4}\right]+{\;e}^{-}⇋{\;C}_{n}+{\;\mathrm{AlCl}}_{4}^{-}\;$$

(3) $$Net\;reaction:\mathrm{Al}+{\mathrm{AlCl}}_{4}^{-}+3{\;C}_{n}\left[{\mathrm{AlCl}}_{4}\right]⇋\;4{\;\mathrm{Al}}_{2}{\mathrm{Cl}}_{7}^{-}+3{\;C}_{n}$$

The majority of research efforts on ABs are mainly focused on the investigation of new cathode materials and electrolytes, to improve performance and reduce costs. Different from Li^+^, Na^+^ and K^+^ ion batteries, in which the carbon material is used as an anode, for ABs, it is employed as a cathode. In 1988, a pioneering work based on graphite as a carbon-based cathode for ABs was published [77] with discharged capacities varied between 35 to 40 mA h g^−1^ when operated at 1.7 V and the current densities of 1–10 mA g^−1^ exhibiting 100% Coulombic efficiency (CE) for 150 cycles but unfortunately, the generation of gas in the cell hindered further development of this system.

Perhaps, one of the most interesting works on carbon cathodes in AIBs is the one reported by Lin et al. in 2015 [66] wherein they used three-dimensional graphite as a cathode that yielded an “ultrafast rechargeable aluminum-ion battery”. The AB cell withstood cycling rates up to 5000 mA g^−1^ without losing its capacity and maintaining a CE > 95%. The cell exhibited well-defined voltage plateaus at around 2.0 V and very good stability over 7500 cycles. However, it showed important drawbacks such as low CE at the slowest charging speeds reported (100 mA g^−1^), and an overall sub-par specific discharge capacity of around 66 mA h g^−1^. The battery cell provided an energy density of 40 W h kg^−1^, which is comparable to lead-acid and Ni-MH batteries, as well as a power density near 3000 W kg^−1^, which is in the range of supercapacitors. These very promising results served as a starting point to be further researched and followed by a series of works aimed at improving on the same concept. Yang et al. [78] addressed the issue of the low specific capacity stated the in the aforementioned study. A 3D graphene was fabricated and employed as an AB cathode, which exhibited outstanding electronic and mechanical features and comparable electrochemical performance to the material synthesized by Lin et al. [66]; however, no remarkable improvements were achieved by this work. Wang et al. [79] employed a cathode based on pure graphite flakes. Despite the simple composition of the cathode, the AB cell showed good electrochemical performance. This work revealed the structural modifications of the cathode provoked by intercalation/deintercalation of AlCl_4_^−^ that seemed to be reversible as deduced from XRD and Raman spectroscopy. These important achievements are related to the charge storage mechanism that is based on intercalation/deintercalation processes of AlCl_4_^−^ in the cathode and conversion reactions of Al anode where three electrons are exchanged for net seven anions that are oxidized or reduced as seen in Equations (1) and (2). Because of that, Al-anode possesses an extremely large theoretical specific capacity (2980 A h kg^−1^). Moreover, the Al^3+^ ionic radius size is 53.5 pm, which is much smaller than Li^+^ (76 pm), something that makes Al^3+^ a much more suitable candidate for the intercalation process on biomass-derived electrodes. Porous carbons as anodes of Al-based DIBs are capable of storing Al/Al-ions by reversible conversion reaction, $Al_{x}+C_{y}⇋\;Al_{x}C_{y}$ [80,81,82]. Such carbon anodes are potential candidates for fetching the concept of AIBs into reality but are largely unexplored.

However, with the recent great progress of ABs, they are plagued by low experimental capacities issues which hinder their commercial application. The low capacity of ABs mainly arises from the larger anionic size of charge carrier AlCl_4_^−^ (6.09 Å) which is twice the *d*-spacing of graphite (3.35 Å). In addition, the most commonly studied cathodes such as graphite and few-layered graphene (FLG) are prone to undergo huge volume expansions (up to 600%) upon AlCl_4_¯ insertion that eventually lead to cathode failure [65,66]. FLG manages such failure but still undergoes continuous inconvenient structural changes over long-term cycling [65]. Therefore, it is obvious to look for alternate AB cathode materials that not only evade existing obstacles of the graphitic cathodes but also promising better performance with higher rate capabilities and long-lasting durability. Biomass-derived activated carbon (AC) materials are of great choice as cathodes for ABs applications. Similar to various surface and 3D engineered graphene materials, AC is also expected to perform better as the cathodes by overcoming the low capacity as well as buffer huge volume change issues of the graphitic cathodes owing to their 3D interconnected networks and high porosity [83].

There are very few reports available regarding the applications of biomass-derived carbons for AB cathodes as listed in Table 3. Das and co-workers stated that the overall mechanism of non-aqueous AB is based on the intercalation of complex tetrachloroaluminate anion (AlCl_4_^−^) [84] and that its reaction refers to the reversible intercalation of ions (anions containing aluminum) into the lattice of the layered cathode (biomass-derived carbon materials) as described in Figure 6 and the reversible half-cell reaction (2). Charge storage still depends on AlCl_4_^−^ storage which generates from conversion between Al and Al_2_Cl_7_^−^ as seen in Figure 6 and half-cell reactions (1) and (2). Thus, naming the electrochemical energy storage devices built with the bio-based carbon cathodes and the IL electrolytes as ABs is meaningful. The lattice spaces and electronic properties of the carbonaceous structures play a huge role in the AlCl_4_^−^ intercalation reaction which in turn provokes important changes in the interlayer spacing during the charging-discharging process. These biomass-derived ACs present very poor graphitic structures which lack regularly ordered carbon positions (parallel layers of graphene sheets) that are weakly bound together by Van der Waals forces [85]. These graphene layers are separated by a distance comparable to the Van der Waals gap (defined as the chalcogen-to-chalcogen distance); i.e., approximately 3–3.5 Å [86], and bigger gaps could lead to a much-expanded lattice that is very suitable for intercalation process of many ions in battery systems such Li^+^, Na^+^, K^+^ and AlCl_4_^−^. Thus, the employment of strategies to increase the graphitization degree in the biochars as well as the Van der Waals gaps and therefore expansion of the carbon lattices are highly required to boost the bio-based anodes/cathodes’ electrochemical properties.

Shkolnikov et al. [87] prepared AC from birch sawdust that performed well as an AB cathode compared to commercial porous carbon, CMK-3 [87,88]. They carbonized the birch sawdust at 400 °C which was later activated with NaOH (4 wt.%) by heating at 700 °C that resulted in AC with a high surface area of 3300 m^2^ g^−1^ (SSA) and 1230 mm^3^ g^−1^ of micropore volume [89]. Upon use as an AB cathode, this AC delivered a discharge capacity of 82 mA h g^−1^. The capacity was retained at 69 mA h g^−1^ (>84%) up to the 40th cycle but it performed inferior to the graphite’s 100% capacity retention (75 mA h/g) throughout the cycling under similar testing conditions. The synthesis approach needs to be optimized further to match the performance of the AC with graphite and for improving its cyclability. One strategy could be to modify the porosity further. Here microporosity observed could be beneficial for an electric double layer of charge carriers (EDLC of AlCl_4_^−^ and Al_2_Cl_7_^−^) similar to supercapacitors but not their channeling (intercalation) often noticed in batteries that are promising for higher capacity as noticed in the graphite [89]. Porosity in the range of meso (from 2 to 50 nm) and macropores (>50 nm) was good for ions transport i.e., intercalation and deintercalation [89]. Hence, engineering the porosity of biobased AC is crucial for better AB performance as reported by Thanwisai et al. in their study with coconut shell-derived AC [76].

**Figure 7:**
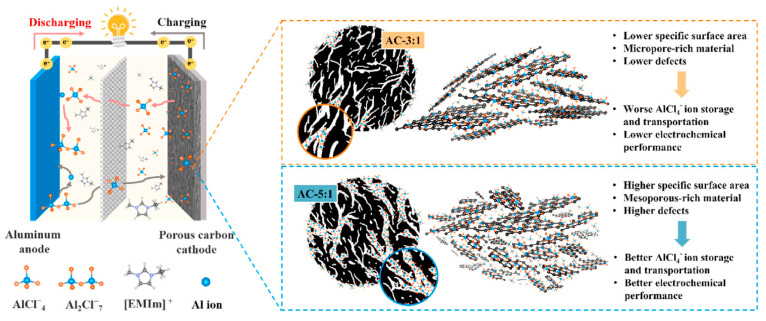
Schematic illustration of AlCl_4_^−^ ion storage by biomass-derived carbon cathodes in a non-aqueous AIB. Reprinted with permission from [76]. Copyright 2022, Elsevier.

Thanwisai et al. [76] reported obtaining an AC with 2686 m^2^ g^−1^ SSA and 1484 cm^3^ g^−1^ pore volume by activating coconut shell chars with KOH in a 1:5 mass ratio, respectively, at 850 °C under an argon atmosphere. The product AC as AB cathode delivered a discharge capacity of 90 mA h g^−1^ at a high current rate of 1.0 A g^−1^ that retained up to 81 mA h g^−1^ (90%) even after 1500 cycles. It is worth noticing that the AC cathode outperformed CMK-3, other than its class of materials as shown in Table 3, and even graphite/FLGs as reported elsewhere [76]. The authors claimed that the high presence of defects, high SSA, and abundance in mesoporous synergically acted to yield cathodes with excellent electrochemical performance as described in Figure 7. Other ACs derived from various sources such as human hair, sucrose, tar pitch, etc. seemed better alternates to CMK-3 but they still lag behind the performance of AC from coconut shells. However, the reports listed in Table 3 did not provide long-term cycling details of various bio-derived ACs, it is evident from their initial cycling trend that an increase in porosity is promisingly reflected in an improved capacity that retains greatly when compared to commercial CMK-3. However, biobased carbons need to perform further better to catch up with commercial graphene cathodes [83]. Therefore, there is still scope to improve the performance of the ACs by opting for different biomass sources, surface modification, functionalization, internal networking, improving inherent electrical conductivity, modifying the porosity beyond mesoporous, changing binder, electrolyte, etc. like those pursued with various reported FLG materials [10,65,83,84,90].

**Table 3:** Comparative electrochemical performance of biobased carbon material anodes for Al battery.

Source	Synthesis Method and Morphology	Specific Surface Area (SSA, m^2^ g^−1^)	Operating Potential (V)	Current Rate (mA g^−1^)	Initial Discharge Capacity (mA h g^−1^)	Capacity Retention (mA h g^−1^)/(Cycles)	Ref.
Sucrose	Sucrose was carbonized over customized silica template particles and washed with NaOH etching. The carbonization was carried out at 900 °C under H_2_/Ar atmosphere.	1185	0.01–2.25	500	82	70 (1000)	[71]
Tar pitch	The reaction mixture of tar pitch and KOH (1:4 wt.%) was heated at 850 °C to produce activated carbon (AC). The porosity of AC is further modified by heat treatment at 750 °C under N_2_ flow.	1980	0.01–2.25	500	51	95 (1000)	[71]
Commercial CMK-3	-	1000–2000	0.5–2.3	980	~27	33 (36,000)	[88]
Birch sawdust	Carbonized birch sawdust (400 °C) activated with 4 wt.% of NaOH under Ar flow at 600–850 °C.	3300	0.0–2.5	283	82	69 (40)	[87]
Coconut shell	coconut shell chars were activated with 5 wt.% of KOH at 850 °C under an argon atmosphere.	2686	0.01–2.2	1000	90	81 (1500)	[76]
Human Hair	Hair samples brunt at 300 °C were activated with 2 wt.% of NaOH at 750 °C in Ar flow.	-	0.2–2.45	50	103	100 (50)	[91]

## 6. Conclusions, Challenges and Future Works

Biomass-based materials have the potential to become the most promising electrode candidates for high-performance batteries due to important advantages such as vast abundance, sustainable approach, and tunable physicochemical nanostructures. Besides, the characteristics/composition of the biomass plays an important role in the carbon electrode properties as well as how they are made (different pyrolysis and activation experimental conditions) which influences their performances as electrodes. As for biomass-derived carbon electrode materials, their suitable electronic conductivity, and tailor-made physicochemical properties such as high surface area and different pore structures, presence of functional groups and elements (O, H, N, S, F, etc) have made them promise and extremely suitable candidates for efficient active electrode materials to carry out and boost electrochemical reactions in KIBs and ABs allowing high-performance battery devices. 

In summary, the development of KIBs anodes and ABs cathodes continues to be a critical barrier for them to achieve higher capacities and therefore impede their commercialization. However, much has been learned about these materials, syntheses processes and innovations, and charge storage mechanisms in the last few years. At this rate, the future of KIB and AIB systems can only get better with time.

Over the last few years, important efforts on developing biomass-derived carbon materials to be employed as electrodes for battery applications have been made. However, considerable practical and technological challenges need to be addressed to overcome issues related to such applications, so we pointed out four key important challenges as follows:

1. Difficulties in selecting proper and suitable biomass and carbon preparation methods to obtain desirable and tailored shapes/pores despite thermochemical routes employed in the conversion of biomass-precursor into carbon electrode materials.
2. Issues on controlling and tailoring the pore geometry and size (pore structure), and interlayer carbon spacing by using chemical activators to obtain high and stable SSA suitable for any kind of battery application.
3. a better understanding of the mechanism(s) and effect(s) of SSA, pore structure, interlayer spacing, and surface chemistry on electrochemical performance in the preparation of high-efficient biomass-carbon electrodes in a battery application.
4. Weak development of more sustainable (e.g., cheaper and environmentally friendly) large-scale synthesis capabilities for biomass-carbon electrodes with suitable structures and properties for commercial battery application.

By overcoming the above challenges, some future works should be addressed as follows:

1. A deeper understanding of the composition of biomass precursors and the biomass-related effects on the properties of the final bio-based carbons.
2. Deepening in the charge storage mechanisms (intercalation versus pseudocapacitance) in connection with textural properties, pore size, distribution, etc.
3. A deeper understanding of the carbon electrode properties on the large volumetric variation induced by large ions (K^+^, AlCl_4_^−^, and Al_2_Cl_7_^−^) size during charge and discharge processes and their sluggish reactions kinetic, so carbon electrodes with outstanding physicochemical features must be developed to overcome these issues.
4. Biomass-derived ACs are potential anode candidates for future battery technology such as ABs. Studies regarding the Al^3+^ storage mechanism and its consequences on the physical, chemical, and physicochemical properties of ACs have not progressed much. Hence we recommend pursuing research in this direction as the next immediate step.

## Figures and Tables

**Table 2:** Comparison of C_g_ and C_v_, standard redox potential (E_o_ vs. SHE), ionic radius (r_ion_), and abundance in the Earth’s crust of various metal anodes [67].

Metal Anode (Counter Electrode)	C_g_ (A h g^−1^)	C_v_ (A h cm^−3^)	E_o_ (V)	r_ion_ (Å)	Abundance (wt.%)
Li^+^/Li	3.86	2.06	−3.0	0.76	0.006
Na^+^/Na	1.17	1.13	−2.7	1.02	2.8
Mg^2+^/Mg	2.20	3.84	−2.4	0.72	2.1
Al^3+^/Al	2.98	8.04	−1.7	0.53	8.1
K^+^/K	0.685	0.61	−2.9	1.38	2.09

## Data Availability

Not applicable.
